# A classification algorithm based on dynamic ensemble selection to predict mutational patterns of the envelope protein in HIV-infected patients

**DOI:** 10.1186/s13015-023-00228-0

**Published:** 2023-06-19

**Authors:** Mohammad Fili, Guiping Hu, Changze Han, Alexa Kort, John Trettin, Hillel Haim

**Affiliations:** 1 Department of Industrial and Manufacturing Systems Engineering, Iowa State University, 3014 Black Engineering, 2529 Union Drive, Ames, IA 50011 USA; 2 Department of Microbiology and Immunology, Carver College of Medicine, University of Iowa, 51 Newton Rd, 3-770 BSB, Iowa City, IA 52242 USA

**Keywords:** Classification algorithm, Dynamic ensemble selection, HIV-1, Virus evolution, K-best classifiers, Protein structure

## Abstract

**Background:**

Therapeutics against the envelope (Env) proteins of human immunodeficiency virus type 1 (HIV-1) effectively reduce viral loads in patients. However, due to mutations, new therapy-resistant Env variants frequently emerge. The sites of mutations on Env that appear in each patient are considered random and unpredictable. Here we developed an algorithm to estimate for each patient the mutational state of each position based on the mutational state of adjacent positions on the three-dimensional structure of the protein.

**Methods:**

We developed a dynamic ensemble selection algorithm designated k-best classifiers. It identifies the best classifiers within the neighborhood of a new observation and applies them to predict the variability state of each observation. To evaluate the algorithm, we applied amino acid sequences of Envs from 300 HIV-1-infected individuals (at least six sequences per patient). For each patient, amino acid variability values at all Env positions were mapped onto the three-dimensional structure of the protein. Then, the variability state of each position was estimated by the variability at adjacent positions of the protein.

**Results:**

The proposed algorithm showed higher performance than the base learner and a panel of classification algorithms. The mutational state of positions in the high-mannose patch and CD4-binding site of Env, which are targeted by multiple therapeutics, was predicted well. Importantly, the algorithm outperformed other classification techniques for predicting the variability state at multi-position footprints of therapeutics on Env.

**Conclusions:**

The proposed algorithm applies a dynamic classifier-scoring approach that increases its performance relative to other classification methods. Better understanding of the spatiotemporal patterns of variability across Env may lead to new treatment strategies that are tailored to the unique mutational patterns of each patient. More generally, we propose the algorithm as a new high-performance dynamic ensemble selection technique.

## Background

Four decades after recognizing human immunodeficiency virus type 1 (HIV-1) as the causative agent of acquired immune deficiency syndrome (AIDS), this virus is still a major health concern worldwide. In the year 2021, 38 million individuals were living with HIV, 650,000 died from AIDS-related diseases, and 1.5 million were newly infected [1]. To treat HIV-infected individuals, multiple therapeutics are available; they bind to the viral proteins and can effectively inhibit their function. However, the replication machinery of HIV-1 is prone to errors. As a result, new variants of its proteins are generated, some of which contain changes at sites targeted by the therapeutics [2]. Subsequent expansion of the mutant forms under the selective pressure of the therapeutic can lead to clinical resistance [3, 4]. Since the appearance of the mutations is random, the emergence of resistance by changes at any position of an HIV-1 protein is considered unpredictable. There is a critical need to better understand the changes in HIV-1 within the host. Such knowledge can lead to the design of new strategies that tailor treatments to infected individuals based on the properties of the infecting virus and the changes expected to occur. Multiple tools have been developed over the past two decades to predict the evolution of other viruses, primarily influenza virus, to inform the design of vaccines according to the changes expected to occur [5]. Unfortunately, the number of tools developed to model and predict the changes in HIV-1, particularly within the host, is limited [6–9].

### Toward a better understanding of variability patterns in the envelope proteins of HIV-1 within the infected host

Of all HIV-1 proteins, the envelope glycoproteins (Envs) exhibit the highest level of diversity, both within and between hosts [10, 11]. Env adorns the surface of HIV-1 particles and allows the virus to enter cells [12]; it is thus a primary target in AIDS vaccine design [13]. Env is composed of approximately 850 amino acids (some diversity in length exists between different strains). In the infected host, new amino acid variants continuously appear at multiple positions of this protein. Consequently, at any time point during chronic infection, 10% or more of Env positions can exhibit variability in amino acid sequence between co-circulating strains [14, 15]. The random nature of the mutations, the extreme diversity of Env within and between hosts, and the structural complexity of this protein limit our ability to model the changes.

Whereas the amino acid that occupies any Env position can vary between strains in different hosts, the level of in-host variability in amino acid sequence at each position shows clear specificity for HIV-1 subtype (clade) [9]. Thus, patterns of variability in the host are not merely random “noise” but reflect inherent properties of the virus. Variability describes the permissiveness of each site to contain amino acids with different chemical properties, which reflects the strength of the selective pressures applied on the site. In this work, we investigated the spatial clustering of variability across the Env protein. Specifically, we tested the hypothesis that the absence or presence of sequence variability at any position of Env can be predicted based on the variability at adjacent positions on the three-dimensional structure of the protein. If the propensity for co-occurrence of a high-variability state at adjacent positions is “stable” over time, then such patterns may capture the likelihood of each position to undergo changes at future time points. To test the above hypothesis, we developed a new algorithm that selects the best subset of classifiers to predict the class label (variability status) of each new observation (patient) using a dynamic mechanism.

### Multiple classifier selection

As the complexity of a dataset increases, the ability of any single classifier to capture all patterns is reduced, requiring integration of multiple classifiers to improve classification accuracy. However, the use of the same set of classifiers statically over the entire feature space can affect the overall performance of an algorithm (i.e. a classifier may perform well in some subspaces of the data but exhibit poor performance in others). One solution to this problem is the dynamic selection of the optimal classifier/s for each new instance from a pool of existing classifiers based on some evaluation criteria, and application of this subset to predict the class label of the instance. Dynamic ensemble selection (DES) techniques apply this approach. They are composed of three steps: (i) Classifier generation, (ii) Ensemble selection, and (iii) Classifier combination.

In the first step, a pool of heterogenous [16–18] or homogenous classifiers [19–21] is generated and then trained on the dataset. Strategies employed in DES methods for training include subspace sampling [22], bagging [23], stratified bagging [24], boosting [25], and clustering [26]. In the second step, ensemble selection, the mechanism to select the best subset of base learners for each prediction is defined. Selection can be based on probabilistic models [27], or by incorporation of multiple classifiers to increase the diversity of the base learners [28]. The third step of DES, classifier combination, aggregates the gathered information into a single class label (prediction). Aggregation methods include Dynamic classifier weighting [29, 30], artificial neural networks (ANNs) [31] and majority voting [32].

### The k-best classifiers (KBC) algorithm

Here we describe a novel algorithm, which utilizes a dynamic mechanism to select the best classifiers for predicting the class label of each new instance. Classifiers are chosen based on their performance in the neighborhood of a new observation (i.e. instances with similar profiles). Bootstrap resampling is used to increase randomness, thus introducing more diversity within the base learners. This creates an out-of-bag sample that can be used along with the resampled data in the classifiers’ evaluation process. We also apply a classifier scoring approach, upon which the selection decision of a classifier is made. To define a neighborhood for a new observation, we use the k-nearest neighbors (KNN) algorithm. The feature vector of each observation is used for the neighborhood selection process. The novelty of this method is in the dynamic classifier selection approach, where we introduce a weighting mechanism to evaluate each classifier’s performance within the neighborhood of a new observation to decide if the classifier contributes to the prediction.

We tested the KBC algorithm with a panel of sequences from HIV-1-infected individuals. These data describe for each patient the absence or presence of variability in amino acid sequence at each position of Env on the three-dimensional structure of the protein. We examined whether the variability at each position (or group of positions) can be predicted based on variability at adjacent positions on the protein. Given the folded structure of the protein, the distance between any two positions (in Ångstroms, Å), as determined by the cryo-electron microscopy (cryo-EM) coordinates of the Env protein, was used as the measure of proximity. In many cases, the KBC algorithm showed higher classification metrics than other machine learning algorithms. Considerably higher performance was observed for the CD4-binding domain of Env, which is the target of multiple antibody therapeutics against HIV-1 [33–37].

## Methods

### HIV-1 Env sequence data

Nucleotide sequences of the HIV-1 *env* gene were downloaded from the National Center for Biotechnology Information (NCBI) database (https://www.ncbi.nlm.nih.gov) and from the Los Alamos National Lab (LANL) database (https://www.hiv.lanl.gov). Sequence data for HIV-1 clades B and C were downloaded and processed separately. The clade C dataset is composed of 1,960 sequences from 109 distinct patients. The clade B dataset is composed of 4,174 sequences from 191 distinct patients. For each patient sample, all Envs isolated were analyzed (at least six sequences per sample). All *env* genes were cloned from the samples by the single genome amplification approach [38] and sequenced by the Sanger method. Sequences of non-functional Envs were removed, as were all sequences with nucleotide ambiguities or large deletions in conserved regions [9, 39]. Nucleotide sequences were aligned using a Hidden Markov Model with the HMMER3 software [40] and then translated into the amino acid sequence, which was used for the analysis. All 856 Env positions described in the manuscript conform to the standard HXBc2 numbering of the Env protein [41]. Potential N-linked glycosylation sites (PNGSs) contain the sequence motif Asn-X-Ser/Thr, where X is any amino acid except Pro. To account for the presence of N-linked glycans on the Asn residues, the first position of all Asn-X-Ser/Thr triplets was assigned a unique identifier. All aligned sequences from each patient were compared to determine whether each of the 856 positions contains variability in amino acid sequence (position is assigned a variability value of 1) or whether all sequences from that patient sample have the same amino acid at the position (assigned a variability value of 0).

### Env structural data

To identify the positions closest to each position of interest, we used the coordinates of the cryo-EM structure of the HIV-1 Env trimer. Coordinates of all three subunits were used in the calculations. For clade B viruses, we used the coordinates of Env from HIV-1 clade B strain JRFL (Protein Data Bank, PDB ID 5FUU) [42]. For clade C viruses, we used the coordinates of Env from HIV-1 clade C strain 426c (PDB ID 6MZJ) [43]. All atoms of the N-linked glycans are associated with the Asn residue at the first position of the PNGS triplet. The distance between any two positions was measured using the coordinates of the closest two atoms of the two amino acids. These data were used to identify the ten closest positions to each position of interest.

### The KBC algorithm

**Fig. 1:**
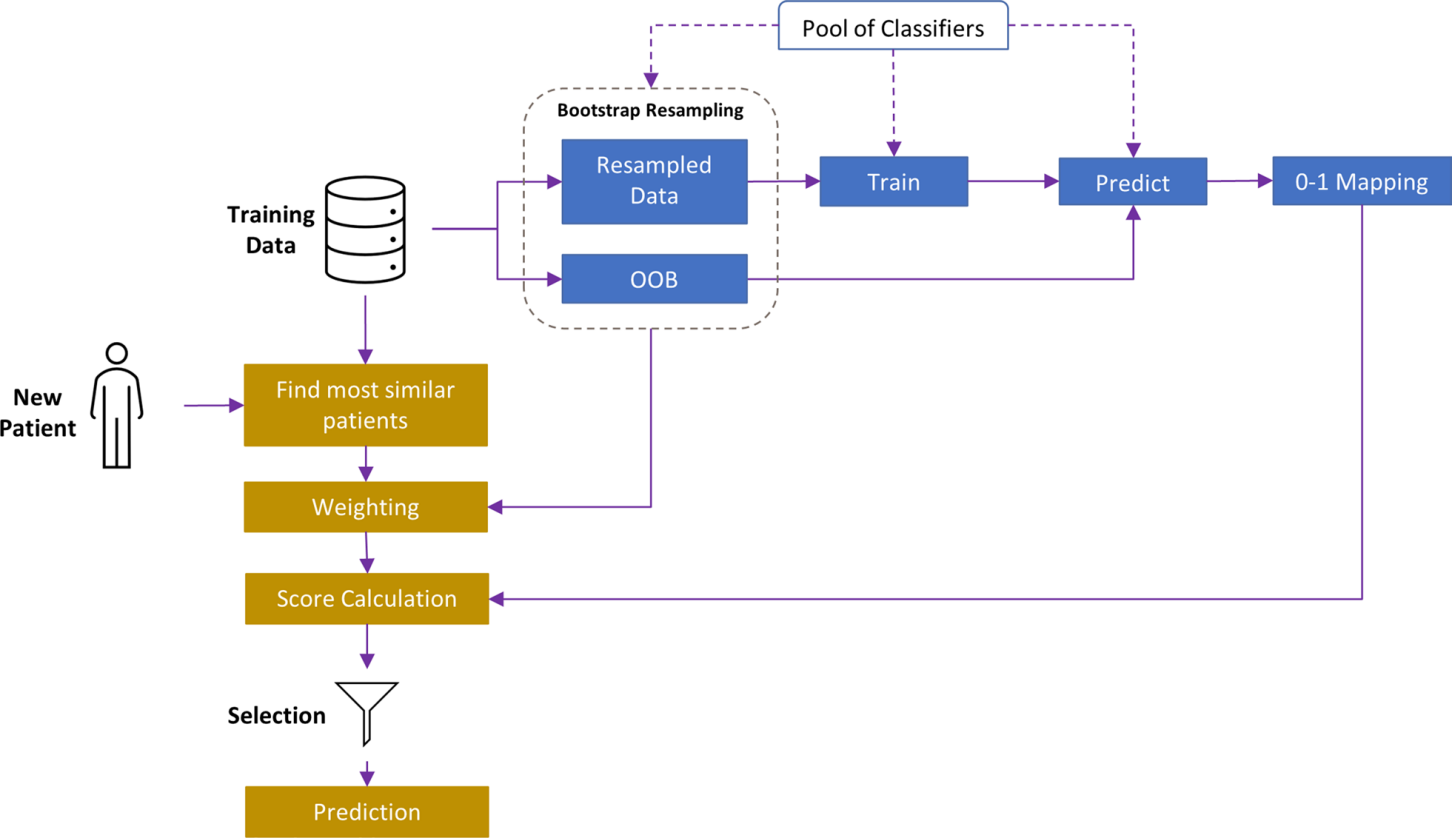
Flow chart of the KBC algorithm. The workflow starts with bootstrap resampling for each base learner. Then, for the neighborhood of each new data point (here, similar patients), weights are assigned to the OOB and resampled sets, and then aggregated into a single score for each learner. Those base learners that surpass the minimum threshold are selected for the prediction of the class label for the new data point

We apply the KBC algorithm to predict the absence or presence of variability at each position of interest in a patient based on the variability at adjacent positions on the protein structure. To this end, KBC applies the information from the training dataset to determine the classifiers that are most helpful in identifying the class label of a new instance based on their performance within a specific neighborhood (i.e., among patients with similar variability profiles in the environment of the site of interest). The foundation of this method, like other dynamic ensemble selection techniques, relies on three main steps: classifier generation, selection, and aggregation. A flow chart of the KBC algorithm is shown in Fig. 1 and further explained below.

1. Classifier generation

We randomly divide our data into a training set (${X}^{train}$) and a test set (${X}^{test}$). The latter is ultimately used for evaluating performance of the algorithm. Then, using ${X}^{train}$ we follow the steps below. First, we generate a pool of $M$ base learners, ${L}_{1}, {L}_{2}, \dots , {L}_{M}$. The number of base learners is a hyperparameter in the KBC algorithm. Here we use decision trees as the base learners, primarily for their training speed. In the next step, we use bootstrap resampling to create two sets of data for each base learner ${L}_{i}$: **(i)** A resampled set or “bag” (denoted as ${S}_{i}^{r}$) which refers to the observations selected through the resampling procedure, and **(ii)** An Out-of-Bag (OOB) set (denoted as ${S}_{i}^{oob}$), which includes the remaining observations not in ${S}_{i}^{r}$. Each base learner ${L}_{i}$ is trained on the corresponding resampled set (${S}_{i}^{r}$) and evaluated using both the resampled and OOB set. Utilization of the unseen OOB set provides a more robust evaluation of the base learner.

We define the training set of samples ${X}^{train}$ with ${x}_{1}, {x}_{2}, \dots , {x}_{N}$ as the observations, and ${y}_{1}, {y}_{2}, \dots , {y}_{N}$ as their corresponding class labels. We also denote the test set as ${X}^{test}$, where:

1 $${X}^{train}={S}_{i}^{r}\cup {S}_{i}^{oob},\quad \forall i=1, 2, \dots , M$$

2 $${S}_{i}^{r} \cap {S}_{i}^{oob}= \varnothing ,\quad \forall i=1, 2, \dots , M$$

If we define an event $A$, as a data point ${x}_{j}$ ($j=1, 2, \dots , N$) that belongs to the OOB sample:

3 $$A: {x}_{j} \in {S}_{i}^{oob},\quad \forall i=1, 2, \dots , M$$

then the probability of such an event can be calculated as:

4 $$Pr\left(A\right)={(1-\frac{1}{N})}^{N}\approx {e}^{-1}\approx 0.368$$

where, $N$ is the total number of observations in the training set ${X}^{train}$. Later, we will show how to use this information in the algorithm as a starting point.

To increase the variability among the base learners, we randomly sample features. For this purpose, the algorithm randomly picks for ${L}_{i}$ a set of $f$ features out of all available features. In other words, the learner ${L}_{i}$ is trained over the subset of the features of ${S}_{i}^{r}$ which is denoted by ${S}_{i}^{r, f}$. Knowing the set of $f$features for the learner ${L}_{i}$, one can also create ${S}_{i}^{oob, f}$ for the evaluation phase.

2. Classifier selection

First, each base learner is used to predict all instances in ${X}^{train}$, including the resampled and OOB data. Then, the classification results are mapped onto a binary variable, ${z}_{ij}$, which is 1 or 0 based on whether the classifier ${L}_{i}$ correctly classified the instance ${x}_{j}$ or not, respectively:

5 $${z_{ij}} = \left\{ {\begin{array}{*{20}{c}} 1&{if\,{{\hat y}_{ij}} = {y_j}} \\ 0&{Otherwise} \end{array}} \right.$$

where, $i=\text{1,2},...,M$ is the base learner index, $j=\text{1,2},\dots ,N$ is the observation index, and ${\widehat{y}}_{ij}$ is the class label that is predicted by the learner ${L}_{i}$ for ${x}_{j}\in {X}^{train}$. The product of this phase is an M*N binary matrix $Z$, in which each row represents the mapped prediction result for one base learner, and each column corresponds to an observation in the training set, ${X}^{train}$ :

6 $$Z={\left[{z}_{ij}\right]}_{M\text{*}N}$$

For efficiency, we perform this only once for all observations rather than during each iteration. In effect, not all observations are used for selecting the best classifiers, but only the ones in the neighborhood of the new observation ${x}_{q}\in {X}^{test}$. To find the neighbors (i.e., the closest data points to the observation of interest), we use the KNN algorithm.

By defining ${\varPsi }_{q}^{n}$ as the neighborhood of a new data point ${x}_{q}$ which includes n-closest observations, we define:

7 $${\phi }_{q}^{n}=\{j: 1\le j\le N ,\quad {x}_{j}\in {\varPsi }_{q}^{n}\}$$

where, ${\phi }_{q}^{n}$ is the set of $n$ indices for the data points within the neighborhood of ${x}_{q}$.

To account for the differences in performance of the base learners for the OOB and resampled sets, we assign greater weights to the observations in the OOB set. Weighting of the OOB and resampled sets can be described by:

8 $${W}^{oob}+{W}^{r}=1$$

9 $${W}^{oob}, {W}^{r}>0$$

where, ${W}^{oob}$ and ${W}^{r}$ are the weights for observations within the OOB (${S}_{i}^{oob,f}$) and resampled (${S}_{i}^{r,f}$) sets for learner ${L}_{i}$, respectively. From Eq. 4, we can conclude that the probability of a data point belonging to ${S}_{i}^{r,f}$ is approximately 0.632. We can use this value as the default ${W}^{oob}$; however, the optimal value for this parameter can be obtained via hyperparameter tuning. In general, the higher the OOB weight, the greater the focus on the OOB observations rather than the resampled set.

Now, consider the matrix $\varPi$ in which the type of data points (i.e., being from the OOB or resampled set) is stored:

10 $$\varPi ={\left[{\pi }_{ij}\right]}_{M*N} , i=\text{1,2},\dots , M \,and\, j=\text{1,2},\dots ,N$$

where ${\pi }_{ij}$ is defined as:

11 $${\pi _{ij}} = \left\{ {\begin{array}{*{20}{c}} {{W^{oob}}}&{{x_j} \in S_i^{oob,f}} \\ {{W^r}}&{{x_j} \in S_i^{r,f}} \end{array}} \right.$$

where, $i=\text{1,2},\dots ,M$ and $j=\text{1,2},\dots ,N$. In the next step, the classifier’s score, ${CS}_{i}$, is calculated for base learner ${L}_{i}$:

12 $${CS}_{i}=\sum _{j\in {\phi }_{q}^{n}}{\pi }_{ij}{z}_{ij}, \quad\forall i=\text{1,2},\dots , M$$

Then, we scale the scores:

13 $$C{S}_{i}^{{\prime }}=\frac{{CS}_{i}-\underset{h}{\text{min}}{CS}_{h}+1}{\underset{h}{\text{max}}C{S}_{h}-\underset{h}{\text{min}}{CS}_{h}+1} , \quad\forall i=\text{1,2},\dots ,M$$

where, $h=\left\{\text{1,2},\dots ,M\right\}$ is the set of all classifiers. This rescales all scores into a range of (0, 1], and facilitates the comparison between the classifiers:

14 $${0<\text{C}S}_{i}^{{\prime }}\le 1, \quad\forall i=\text{1,2},\dots ,M$$

Here, ${CS}_{i}^{{\prime }}$ quantifies the relative importance of base learner ${L}_{i}$ to the best classifier.

Next, we consider the relationship between the range of scores assigned by the different classifiers and the normalized scores. As the difference between the performance of the best and worst classifiers increases, there is greater confidence that classifiers with higher scores are performing significantly better than those with lower scores. As shown in Eq. 15, for the extreme case where the range of scores approaches infinity, the difference between the best and worst scaled score converges to the maximum value of 1:

15 $$\underset{{Range}\to \infty }{\text{Lim}}\left( \underset{i}{\text{max}}\left(C{S}_{i}^{{\prime }}\right)- \underset{i}{\text{min}}\left(C{S}_{i}^{{\prime }}\right) \right) \to 1$$

On the other hand, if the range of scores is 0 (i.e., all classifiers have the same performance), the scaled scores will be 1 for all classifiers (no distinction).

Finally, we consider a minimum acceptance threshold ($\delta$) for the classifiers. For different observations, we expect to obtain different arrays of scores for the base learners’ performance within the neighborhoods. Therefore, the algorithm selects the best classifiers by comparison of the score arrays with the threshold $\delta$, considering the problem space, observations, and the base learners’ capabilities to correctly classify similar instances each time.

In Eq. 16, the index corresponding to the k-best classifiers (out of $M$ existing classifiers) for predicting the class label of ${x}_{q}$, is defined as:

16 $${K}_{q}=\left\{i:{ CS}_{i}^{{\prime }}\ge \delta , 1\le i\le M\right\}$$

The number of best classifiers can differ from observation to observation. However, for similar points (i.e., observations within a similar segment of the problem space), we expect to obtain a similar set of best classifiers for prediction.

3. Classifier aggregation

Once the best classifiers for the prediction are identified, we apply an aggregation method to obtain a single result for the new instance. Here we use the majority vote approach. For a general case in which we have $P$ classes, we can write:

17 $${\widehat{y}}_{q}=\underset{p}{\text{Argmax }}\left\{{c}_{p}\right\} , \quad p=\{\text{1,2},\dots ,P\}$$

where, ${\widehat{y}}_{q}$ is the predicted class of the new observation ${x}_{q}$, and ${c}_{p}$ counts the number of base learners predicting class $p$. We can write this as:

18 $${c}_{p}=\sum _{i\in {K}_{q}}{1}_{\left\{{\widehat{y}}_{iq}=p\right\}} ,\quad \forall p \in \{\text{1,2},\dots ,P\}$$

where, ${{\widehat{y}}_{iq}}^{ }$is the class label predicted by learner ${L}_{i}$ for ${x}_{q}$.

### Hyperparameters

The hyperparameters for the KBC algorithm are designed to accommodate the variability in the dataset to ensure maximal performance (see Table 1). $M$ is the number of base learners; if sufficient diversity exists within the base learners (i.e., among the decision trees generated), more learners typically lead to better results. We can also tune the number of features ($f$) for each classifier. Using all available features for each of the base learners can result in lower diversity among the base learners. On the other hand, using too few features, such as the extreme case of $f$=1, can result in a naïve learner that may not be much better than the random guess. By using a suitable fraction of the available features for training the classifiers, we can add variability between the classifiers and increase the confidence that each classifier will perform well.

**Table 1:** Hyperparameters of the KBC algorithm

parameter	Domain	Description
$M$	$\in \mathbb{N}$	Number of initial base learners
$f$	$\in \mathbb{N}$	Number of features to be selected randomly for each base learner
$n$	$\in \mathbb{N}$	Number of close neighbors to a new instance (similar instances)
${W}^{oob}$	$\left[\text{0,1}\right]$	Weight of OOB instances (default = 0.632).
$\delta$	$\left[\text{0,1}\right]$	Minimum acceptance threshold for a base learner’s score to be selected in a neighborhood of a new data point.

The third hyperparameter is the number of neighbors for a new instance ($n$). Increasing the number of neighbors to all training observations ($N$) will lead to the majority vote for a fixed set of classifiers. In this case, we expect to obtain no variance but high bias. At the other extreme, if we use only one neighbor, the variance will be high. Therefore, it is a bias-variance tradeoff, and selecting the optimal $n$ is essential for performance. The effect of this parameter on the accuracy of the model is explored in this study.

The weight of OOB instances (${W}^{oob}$) plays an important role in emphasizing the unseen data for selecting the best classifiers. Since the data in the OOB set are not used during the training of the base learners, predicting them correctly is more important than the observations in the resampled set. Choosing the weight as 1 will completely ignore the resampled data, whereas a weight of 0 will result in using only the resampled data in the training phase. The optimized value for the OOB weight can be obtained by hyperparameter tuning.

The last hyperparameter of the KBC algorithm is the minimum acceptance threshold ($\delta$), which determines the sensitivity in selecting the best classifiers. The higher the threshold, the smaller the number of learners we expect to obtain. In such a case, the variance may increase; however, at the same time, the confidence in the set of selected classifiers in that region increases. By contrast, the use of lower thresholds may result in more learners, which reduces variance.

We note that in the KBC algorithm, one can use any set of homogenous or heterogeneous base learners. Since we use decision tree as the base learner, we add the maximum depth of the tree to the set of existing hyperparameters and tune KBC for the best performance.

**Fig. 2:**
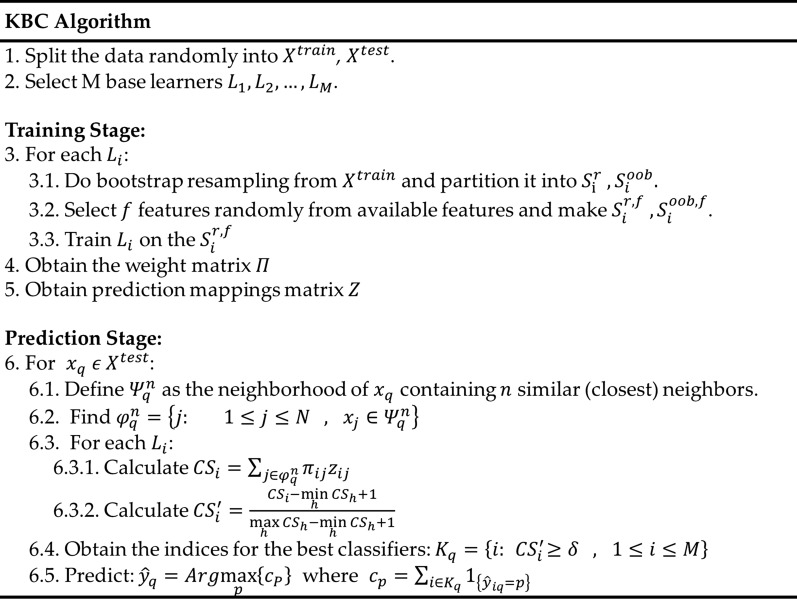
Pseudocode for the KBC algorithm

To summarize the KBC algorithm, we start with the training set from which we create a resampled set and an OOB set for each base learner. The classifiers are then trained separately on their own resampled data. In the prediction phase, for each new data point, we first identify the closest neighbors (i.e., similar patient profiles). Then, based on whether a point belongs to the OOB or resampled set, the method assigns weights to the binary mapping of the initial predictions (1 for correctly classifying an observation and 0 for misclassifying it). This approach introduces a scoring function that is used for evaluating the classifiers. Finally, according to a minimum threshold acceptance value, only those classifiers for which the scaled score exceeds the selected limit is chosen for classifying the new instance. The individual predictions are then aggregated into a single result by the majority vote aggregation method. The pseudocode for the entire KBC algorithm is shown in Fig. 2.

### Evaluation procedure

The ${X}^{test}$ set is used to determine performance of the model generated using the ${X}^{train}$ set. For cross validation, we repeat the random partition of the dataset into ${X}^{train}$ and ${X}^{test}$ five times. To evaluate performance, for any position of Env, we distinguish between two outcome states (class labels): (i) Variability-positive (at least two amino acids are identified at the position in the patient), and (ii) No-variability (all sequences from the patient have the same amino acid at the position). As classification metrics, we use accuracy, precision, recall, F1 score, and balanced accuracy. Accuracy depicts the percentage of predictions that are correct. Precision describes the percentage of correct classifications from the group of instances that are predicted as the positive group. Recall or sensitivity represents the correct classification rate from the group of true positive instances. The F1 score is the harmonic mean of precision and recall. Since the HIV-1 datasets are not balanced (i.e., for any position, the proportion of variability-positive and no-variability samples is not equal), we also use balanced accuracy, which is an average of sensitivity and specificity.

## Results

### Prediction of variability patterns in HIV-1 Env

New forms of the Env protein are continuously generated in HIV-infected individuals by the error-prone replication machinery of this virus. Substitutions at Env positions targeted by therapeutics can lead to virus resistance to their effects. Such events appear to be random and are thus considered unpredictable. There is a clinical need to understand the spatiotemporal patterns of Env variability in the HIV-infected host, which may lead to development of new treatment strategies. We hypothesized that at any time in the infected host, positions that exhibit variability in amino acid sequence are spatially clustered on the Env protein. Such patterns are intuitive since immune and fitness pressures mostly act on multi-position domains of Env rather than individual positions. Toward a better understanding of such patterns, we sought to determine whether the presence of variability at any Env position can be accurately estimated based on the variability at adjacent positions on the protein.

To this end, we tested the KBC algorithm with patient-derived datasets. We used sequence data from 300 patients infected by the two major HIV-1 clades (a total of 6134 sequences). HIV-1 clade C is the most prevalent subtype worldwide and accounts for 46% global infections. HIV-1 clade B is the dominant subtype found in the United States and Europe, infecting more than 90% of all HIV-1 patients in these regions. Given the divergence of the *env* gene between HIV-1 clades B and C, datasets from these two clades were tested separately. We examined the ability of the algorithm to predict the absence or presence of variability at any position ${A}_{p}$ of Env based on the variability at the 10 closest positions on the three-dimensional structure of the protein. Env sequences cloned from patient blood samples were applied (at least six Envs sequenced for each sample). Sequences from each patient sample were aligned and compared to determine the absence or presence of in-host variability at each of the 856 positions of this protein. The response variable is thus the absence or presence of variability at each position ${A}_{p}$. The features are the variability values at the 10 positions closest to position ${A}_{p}$ on the protein, as determined by the physical distance between the closest atoms of the two positions (measured in Ångtroms) on the Env trimer structure. The goal is to correctly classify the variability at position ${A}_{p}$ by the variability profile at adjacent positions. We decided to use the 10 closest positions since this approximates the maximal number of amino acids that can contact the position of interest on the protein structure. We note that the actual number of residues that are in contact with or adjacent to each position may vary according to the location on the protein. For example, for any position buried within the core of the protein, its 10 nearest positions will be closer than for a position located on a loop that is exposed to the solvent. Nevertheless, we decided that as a first step, we will maintain this variable constant for all positions.

**Fig. 3:**
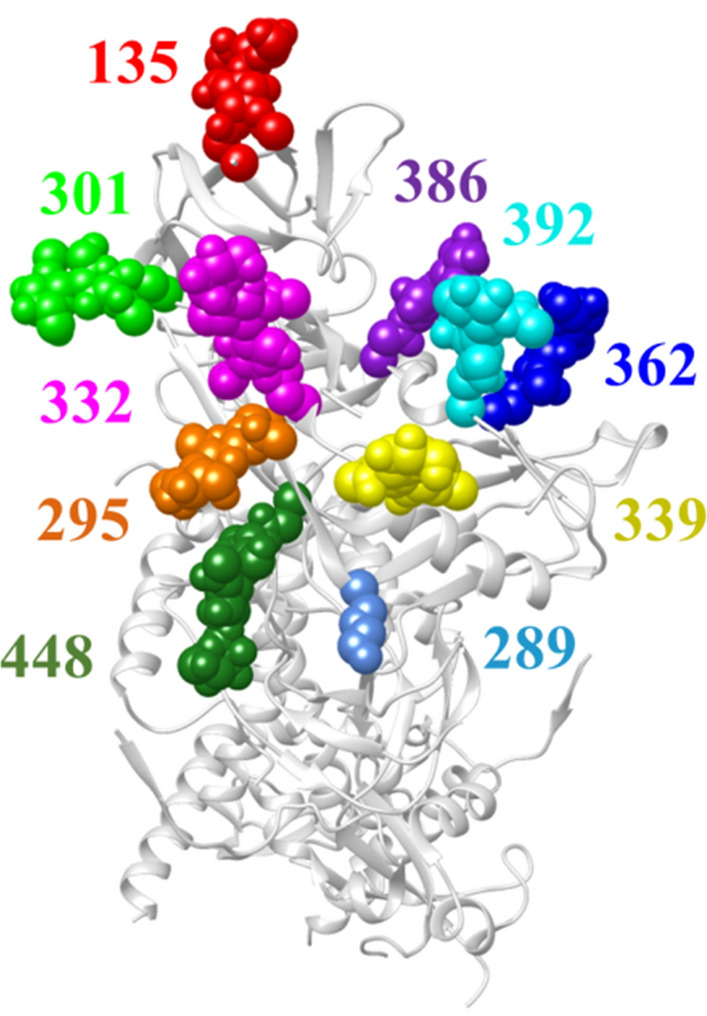
Cryo-EM structure of HIV-1 Env showing positions in the high-mannose patch (PDB ID 5FUU). Positions occupied by glycans are shown as spheres and labeled by Env position number. All positions shown contain N-linked glycans, except position 289, which contains Arg in the Env of HIV-1 isolate JRFL used to generate this structure

We first tested the ability of the KBC algorithm to predict the absence or presence of variability at individual positions in the high-mannose patch of Env (Fig. 3). These N-linked glycans help to shield Env from recognition by host antibodies [44]; however, they also serve as targets for microbicidal agents such as lectins [45, 46] and therapeutic antibodies [47, 48]. We tested three positions in the high-mannose patch, namely positions 289, 332, and 339. These positions form part of the target sites for multiple agents that inhibit HIV-1, including antibodies 2G12, 10-1074, PGT135, PGT128, and DH270.5 [49–53], and the lectin microbicide griffithsin [54]. Data were composed of 1,960 amino acid sequences from 109 patients infected by HIV-1 subtype C, which is the most prevalent HIV-1 clade worldwide [55]. For position 289, the ratio of the variability-positive class to the no-variability class was 34:75. This ratio for positions 332 and 339 was 46:63 and 53:56, respectively.

**Fig. 4:**
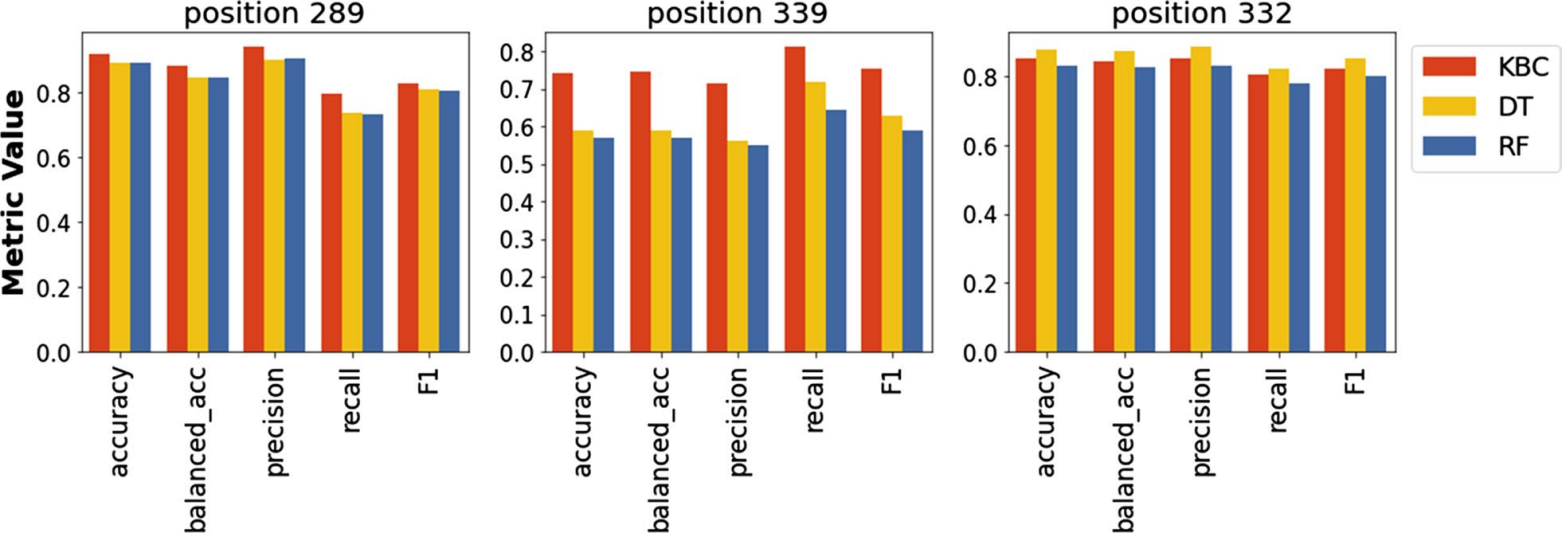
Predictions of variability at positions 289, 339, and 332 of the high-mannose patch. Data describe the results obtained for patients infected by HIV-1 clade C

For positions 289 and 339, the results of the KBC analyses showed improvement relative to the base learner (decision tree) and random forest (Fig. 4**)**. By contrast, the prediction of variability at position 332 by the KBC algorithm was similar to that of the other methods. We also compared the performance of KBC with other machine learning algorithms (Table 2). Again, we observed modestly better performance of KBC for positions 289 and 339, whereas, for position 332, the performance was similar to (or slightly worse than) other methods. We note that although KBC generally exhibited better point estimates than other methods, it also exhibited a relatively high standard deviation (see values in parentheses in Table 2). This likely occurred due to the relatively small size of the dataset. Below, we show that increasing the size of the dataset drastically reduces the standard deviation of the estimates.

**Table 2:** Prediction of variability at Env positions in the high-mannose patch by KBC and other algorithms

Position	Method ^a^	Balanced Accuracy ^b,c^	Accuracy	Precision	Recall	F1 Score
289	KBC	**0.88** (± 0.13)	**0.92** (± 0.08)	**0.94** (± 0.07)	**0.80** (± 0.26)	0.83 (± 0.20)
	QDA	0.83 (± 0.01)	0.88 (± 0.01)	0.90 (± 0.07)	0.71 (± 0.05)	0.79 (± 0.01)
	LDA	0.87 (± 0.02)	0.91 (± 0.01)	0.93 (± 0.05)	0.76 (± 0.05)	**0.84** (± 0.03)
	NB	0.71 (± 0.18)	0.69 (± 0.26)	0.66 (± 0.26)	0.76 (± 0.05)	0.67 (± 0.16)
	ADA	0.86 (± 0.01)	0.90 (± 0.01)	0.91 (± 0.07)	0.76 (± 0.05)	0.83 (± 0.02)
	LogReg	0.86 (± 0.01)	0.90 (± 0.01)	0.91 (± 0.07)	0.76 (± 0.05)	0.83 (± 0.02)
	SVM	0.83 (± 0.01)	0.88 (± 0.01)	0.90 (± 0.07)	0.71 (± 0.05)	0.79 (± 0.01)
339	KBC	**0.75** (± 0.12)	**0.74** (± 0.12)	0.71 (± 0.12)	0.81 (± 0.13)	**0.75** (± 0.11)
	QDA	0.59 (± 0.07)	0.59 (± 0.07)	0.56 (± 0.09)	0.72 (± 0.09)	0.63 (± 0.07)
	LDA	0.57 (± 0.03)	0.57 (± 0.03)	0.55 (± 0.05)	0.64 (± 0.13)	0.59 (± 0.06)
	NB	0.65 (± 0.08)	0.65 (± 0.07)	0.64 (± 0.00)	0.68 (± 0.16)	0.66 (± 0.19)
	ADA	0.60 (± 0.02)	0.60 (± 0.02)	0.59 (± 0.03)	0.62 (± 0.06)	0.59 (± 0.02)
	LogReg	0.59 (± 0.06)	0.58 (± 0.07)	0.54 (± 0.05)	**0.94** (± 0.05)	0.69 (± 0.03)
	SVM	0.65 (± 0.04)	0.66 (± 0.05)	**1.00** (± 0.09)	0.30 (± 0.04)	0.48 (± 0.02)
332	KBC	0.85 (± 0.07)	0.85 (± 0.07)	0.86 (± 0.12)	0.80 (± 0.08)	0.83 (± 0.08)
	QDA	0.84 (± 0.05)	0.84 (± 0.06)	0.84 (± 0.12)	0.83 (± 0.11)	0.82 (± 0.06)
	LDA	0.87 (± 0.03)	0.88 (± 0.03)	**0.89** (± 0.05)	0.83 (± 0.06)	0.85 (± 0.04)
	NB	0.61 (± 0.16)	0.57 (± 0.21)	0.59 (± 0.23)	**0.91** (± 0.13)	0.67 (± 0.10)
	ADA	0.87 (± 0.03)	0.88 (± 0.03)	0.89 (± 0.05)	0.83 (± 0.06)	0.85 (± 0.04)
	LogReg	0.82 (± 0.09)	0.81 (± 0.12)	0.80 (± 0.18)	0.87 (± 0.11)	0.81 (± 0.08)
	SVM	**0.88** (± 0.02)	**0.89** (± 0.02)	**0.89** (± 0.05)	0.85 (± 0.03)	**0.87** (± 0.03)

^a^Calculations were performed using data from 109 patients infected by HIV-1 clade C

^b^Standard deviation values are indicated in parentheses

^c^Values in bold font indicate the highest point estimation value for each metric

Antiviral therapeutics bind to targets composed of multiple residues; their “footprint” on the viral protein can span a large surface that contains multiple amino acids [56–59]. Changes at any of these contacts may reduce Env recognition by the therapeutic and cause resistance. We examined the performance of the KBC algorithm to predict variability in a combined feature composed of 10 positions in the high-mannose patch shown in Fig. 3. To this end, for each position ${A}_{p}$ in the high-mannose patch ($p=1, 2, \dots , 10)$, we relabeled its 10 adjacent positions as ${v}_{\left(1\right)}^{{A}_{p}}, {v}_{\left(2\right)}^{{A}_{p}}, \dots , {v}_{\left(10\right)}^{{A}_{p}}$, where ${v}_{\left(l\right)}^{{A}_{p}}$ is the variability at the $l$-th adjacent position to ${A}_{p} (l=1, 2, \dots , 10)$. For each $l$, we then combined the ${v}_{\left(l\right)}^{{A}_{p}}$ values of the 10 ${A}_{p}$ positions.

We first used the dataset of sequences from 109 HIV-1 clade C-infected individuals. Results were compared between KBC and the above machine learning methods. The ratio of positive-variability to no-variability instances for this dataset was 450:640. Remarkably, KBC performed better than all models to predict sequence variability in the high-mannose patch **(**Table 3**)**.

**Table 3:** Prediction of variability in the high-mannose patch of Env by KBC and other algorithms

Clade	Method	Balanced Accuracy ^a,b^	Accuracy	Precision	Recall	F1 Score
Clade C	KBC	**0.65** (± 0.05)	**0.69** (± 0.05)	**0.67** (± 0.08)	0.47 (± 0.05)	0.55 (± 0.08)
	DT	0.59 (± 0.05)	0.63 (± 0.07)	0.54 (± 0.07)	0.39 (± 0.21)	0.43 (± 0.17)
	RF	0.59 (± 0.02)	0.63 (± 0.03)	0.60 (± 0.05)	0.34 (± 0.13)	0.42 (± 0.10)
	QDA	0.60 (± 0.02)	0.63 (± 0.04)	0.57 (± 0.02)	0.39 (± 0.16)	0.45 (± 0.12)
	LDA	0.58 (± 0.04)	0.62 (± 0.06)	0.54 (± 0.07)	0.39 (± 0.17)	0.44 (± 0.13)
	NB	0.61 (± 0.06)	0.63 (± 0.08)	0.54 (± 0.08)	0.52 (± 0.21)	0.52 (± 0.14)
	ADA	0.50 (± 0.05)	0.44 (± 0.04)	0.42 (± 0.02)	**0.88** (± 0.09)	**0.56** (± 0.02)
	LogReg	0.57 (± 0.06)	0.61 (± 0.04)	0.55 (± 0.07)	0.33 (± 0.20)	0.38 (± 0.15)
	SVM	0.58 (± 0.04)	0.62 (± 0.06)	0.56 (± 0.06)	0.35 (± 0.23)	0.40 (± 0.17)
Clade B	KBC	0.65 (± 0.02)	**0.74** (± 0.02)	**0.68** (± 0.06)	0.39 (± 0.03)	0.50 (± 0.04)
	DT	0.60 (± 0.05)	0.70 (± 0.02)	0.61 (± 0.03)	0.29 (± 0.16)	0.36 (± 0.16)
	RF	0.62 (± 0.00)	0.72 (± 0.00)	0.61 (± 0.02)	0.35 (± 0.02)	0.45 (± 0.01)
	QDA	0.63 (± 0.01)	0.73 (± 0.01)	0.64 (± 0.03)	0.36 (± 0.02)	0.46 (± 0.02)
	LDA	0.64 (± 0.01)	0.72 (± 0.01)	0.61 (± 0.01)	0.40 (± 0.04)	0.48 (± 0.03)
	NB	**0.67** (± 0.04)	0.70 (± 0.03)	0.53 (± 0.05)	0.59 (± 0.07)	**0.56** (± 0.06)
	ADA	0.41 (± 0.05)	0.29 (± 0.02)	0.28 (± 0.03)	**0.74** (± 0.14)	0.40 (± 0.05)
	LogReg	0.64 (± 0.02)	0.72 (± 0.01)	0.61 (± 0.02)	0.39 (± 0.05)	0.47 (± 0.03)
	SVM	0.63 (± 0.02)	0.70 (± 0.02)	0.55 (± 0.03)	0.41 (± 0.01)	0.47 (± 0.02)

^a^Standard deviation values are indicated in parentheses

^b^Values in bold font indicate the highest point estimation value for each metric

To validate these results, we examined the ability of KBC to predict variability in a second panel of sequences derived from individuals infected by HIV-1 clade B. This clade is the most prevalent in the United States and Europe [55]. Sequences from 191 patients were tested to predict variability at the multi-position high-mannose patch using the different algorithms. Consistent with the data shown for clade C, the performance of KBC was superior, albeit modestly, to that of the other algorithms (Table 3). The ratio of the positive-variability class to the no-variability class for the clade B dataset was 621:1289.

**Fig. 5:**
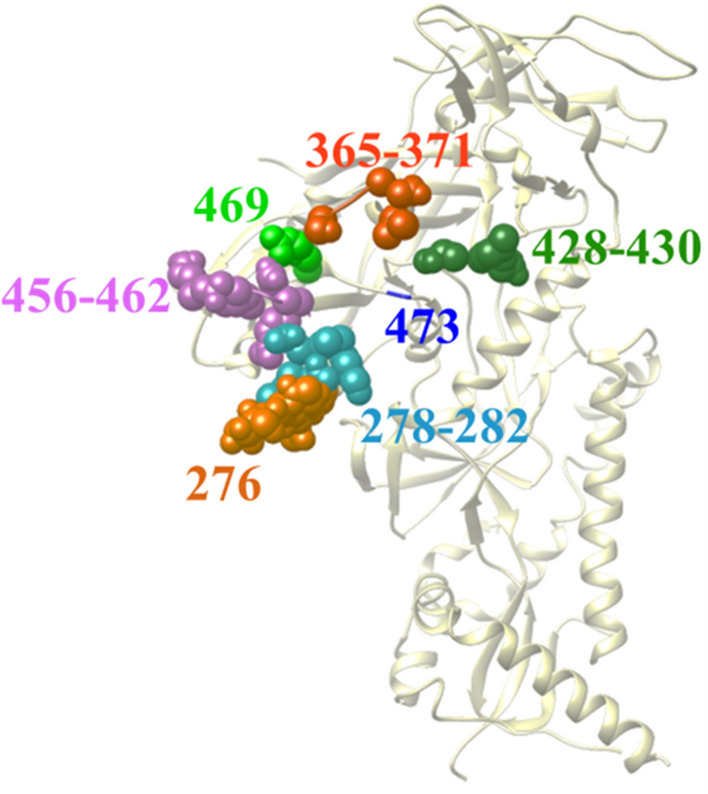
Cryo-EM structure of HIV-1 Env showing positions in the CD4-binding site (PDB ID 5FUU). Positions contacted by antibodies 3BNC117 and VRC01 are shown as spheres and labeled

We expanded our studies to test a second clinically significant domain of the Env protein, namely the CD4-binding site. This domain interacts with the CD4 molecule, which allows entry of the viral genome into the cell [60]. Since this site is conserved among diverse HIV-1 strains, it also serves as a target for multiple therapeutics, including the small molecule Fostemsavir [33] and antibody therapeutics VRC01 and 3BNC117 [34, 35]. We tested a combination of the 23 positions that serve as the contact sites for both antibodies VRC01 and 3BNC117 (Fig. 5). We applied the same procedure explained for the high-mannose patch positions to combine the positions of the CD4-binding site. The ratio of positive-variability to the no-variability classes for the CD4-binding site dataset was 685:3708 and 557:1950 for clades B and C, respectively. The performance of KBC was compared with all other algorithms tested above. Interestingly, the performance of the KBC algorithm was considerably higher than that of other algorithms (Table 4).

**Table 4:** Prediction of variability in the CD4-binding site of Env using KBC and other algorithms

Clade	Methods	Balanced Accuracy ^a,b^	Accuracy	Precision	Recall	F1 Score
Clade C	KBC	**0.71** (± 0.01)	**0.85** (± 0.01)	**0.81** (± 0.07)	0.45 (± 0.03)	**0.58** (± 0.02)
	DT	0.61 (± 0.13)	0.76 (± 0.03)	0.32 (± 0.23)	0.34 (± 0.42)	0.25 (± 0.25)
	RF	0.56 (± 0.04)	0.76 (± 0.03)	0.49 (± 0.08)	0.19 (± 0.18)	0.22 (± 0.14)
	QDA	0.59 (± 0.07)	0.75 (± 0.04)	0.50 (± 0.08)	0.29 (± 0.28)	0.28 (± 0.15)
	LDA	0.69 (± 0.11)	0.79 (± 0.03)	0.59 (± 0.09)	0.50 (± 0.31)	0.46 (± 0.19)
	NB	0.67 (± 0.09)	0.73 (± 0.09)	0.45 (± 0.11)	**0.58** (± 0.31)	0.45 (± 0.16)
	ADA	0.46 (± 0.18)	0.62 (± 0.24)	0.44 (± 0.29)	0.15 (± 0.10)	0.20 (± 0.14)
	LogReg	0.65 (± 0.12)	0.79 (± 0.01)	0.56 (± 0.04)	0.39 (± 0.33)	0.38 (± 0.21)
	SVM	0.64 (± 0.11)	0.76 (± 0.03)	0.50 (± 0.06)	0.43 (± 0.35)	0.37 (± 0.16)
Clade B	KBC	**0.69** (± 0.02)	**0.89** (± 0.01)	**0.78** (± 0.02)	0.40 (± 0.04)	**0.53** (± 0.04)
	DT	0.54 (± 0.02)	0.82 (± 0.02)	0.33 (± 0.02)	0.13 (± 0.08)	0.17 (± 0.10)
	RF	0.53 (± 0.02)	0.82 (± 0.03)	0.35 (± 0.18)	0.11 (± 0.05)	0.15 (± 0.06)
	QDA	0.56 (± 0.03)	0.82 (± 0.04)	0.42 (± 0.16)	0.17 (± 0.11)	0.21 (± 0.09)
	LDA	0.58 (± 0.05)	0.82 (± 0.01)	0.38 (± 0.05)	0.24 (± 0.13)	0.28 (± 0.10)
	NB	0.64 (± 0.08)	0.75 (± 0.10)	0.34 (± 0.14)	0.47 (± 0.21)	0.37 (± 0.13)
	ADA	0.48 (± 0.03)	0.17 (± 0.02)	0.15 (± 0.01)	**0.93** (± 0.10)	0.26 (± 0.02)
	LogReg	0.56 (± 0.04)	0.84 (± 0.00)	0.42 (± 0.05)	0.17 (± 0.10)	0.23 (± 0.11)
	SVM	0.55 (± 0.04)	0.82 (± 0.03)	0.35 (± 0.15)	0.17 (± 0.11)	0.21 (± 0.10)

^a^Standard deviation values are indicated in parentheses

^b^Values in bold font indicate the highest point estimation value for each metric

For positions in the CD4-binding site, the increase in performance was greater than that observed for positions in the high-mannose patch (Table 3). Comparing the results in Tables 3 and 4 shows that the standard deviation of the estimates was considerably lower when we analyzed a group of positions rather than individual positions. For the CD4-binding site, the standard deviation in accuracy, balanced accuracy, recall, and F1 score obtained by KBC was the smallest among all other models for clade C. Indeed, KBC shows higher point estimates as well as smaller standard deviation values for the estimates.

Taken together, these findings show that when Env positions are tested individually, KBC outperforms other algorithms for most (but not all) positions. Nevertheless, this algorithm shines in its performance when tested with a combination of positions that describe the complex (multi-position) target sites of therapeutics on the Env protein.

### Hyperparameter analysis

We examined the effects of two critical hyperparameters of the KBC algorithm on its performance, namely the minimum acceptance threshold ($\delta$) and neighborhood size ($n$). Data that describe variability patterns in the high-mannose patch were used. To evaluate performance, we used the balanced accuracy metric. We explored the effect of one hyperparameter while maintaining the rest at a constant level. We used 20 decision trees ($M$=20); for each, we picked four features randomly ($f$=4), and the maximum depth of the trees was set to be 4. The OOB weight was fixed for both experiments at its default value of 0.632.

**Fig. 6:**
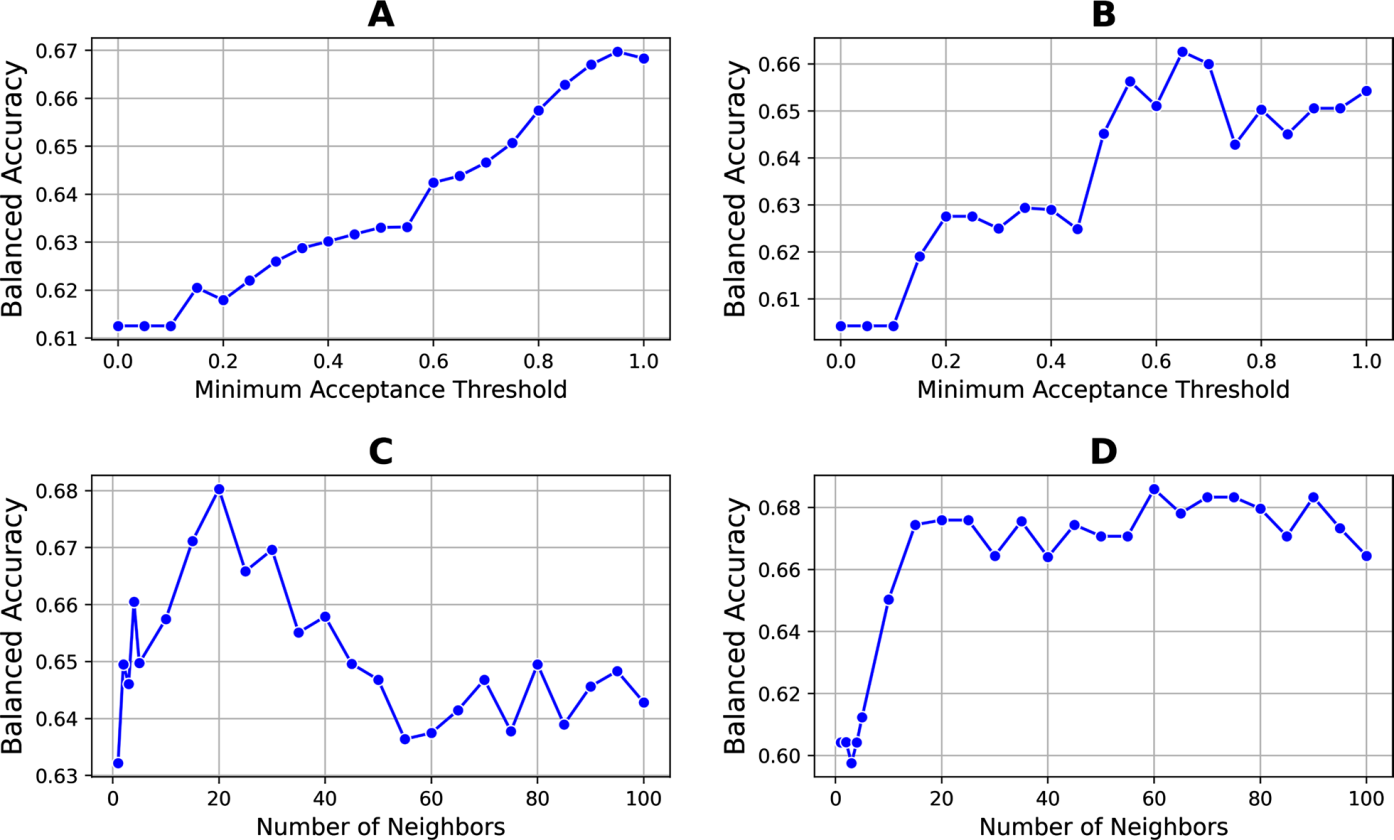
Effect of critical hyperparameters on performance of the KBC algorithm. (**A**, **B**) Effect of the minimum acceptance threshold on balanced accuracy of the algorithm using data that describe variability patterns in the high-mannose patch of clade B and clade C strains, respectively. (**C**, **D**) Effect of the neighborhood size on balanced accuracy of the algorithm using data that describe variability patterns in the CD4-binding site of clade B and clade C strains, respectively

First, we explored the effect of the minimum acceptance threshold ($\delta$). For this experiment, the number of neighbors was set to 10. The experiment was conducted with a variety of thresholds from 0 to 1, and the balanced accuracy was calculated. We observed that for the clade B dataset, increasing the minimum acceptance threshold improved the performance of the KBC algorithm (Fig. 6A). For the clade C dataset, the performance also increased gradually; however, it peaked at a threshold of 0.65, followed by a modest reduction (Fig. 6B). These findings suggest that increasing the value of $\delta$ results in an overall increase in performance due to the higher confidence in the set of selected classifiers. However, in some cases, further increases in $\delta$ may result in the loss of useful classifiers, which can reduce overall performance.

We also explored the effect of neighborhood size on performance of the KBC algorithm. Here we used $\delta$=0.8 as the minimum acceptance threshold. Different numbers of neighbors (ranging between 1 and 100) were tested. We observed that for both clades B and C, increasing the number of neighbors up to approximately 15 or 20 increased the performance (Fig. 6, C, and D). Further increases in the neighborhood size decreased the performance in clade B, whereas it did not impact clade C. These findings suggested that a neighborhood size of approximately 15 is optimal for the data that describe variability patterns in high-mannose patch given the above hyperparameters.

### Effect of base learners on performance of the KBC algorithm

As a further analysis, we examined if the choice of base learners in the KBC algorithm affects the overall performance of the method. To this end, we also tested logistic regression and Naïve Bayes (separately) as the base learners. We evaluated KBC using data from HIV-1 clade C that describe variability patterns in the high-mannose patch and the CD4-binding site. These results were compared with the results obtained using decision tree as the base learner. In this experiment, we maintained the structure of the KBC algorithm as before, with the exception that for each trial, a homogenous set of base learners from one type was utilized (i.e., logistic regression or Naïve Bayes). For the tuning process and for the KBC with logistic regression as the base learner, we incorporated the hyperparameter $C$, which is the inverse of the regularization strength. For the KBC model with Naïve Bayes as the base learner, no hyperparameter was added to the hyperparameters’ list.

**Fig. 7:**
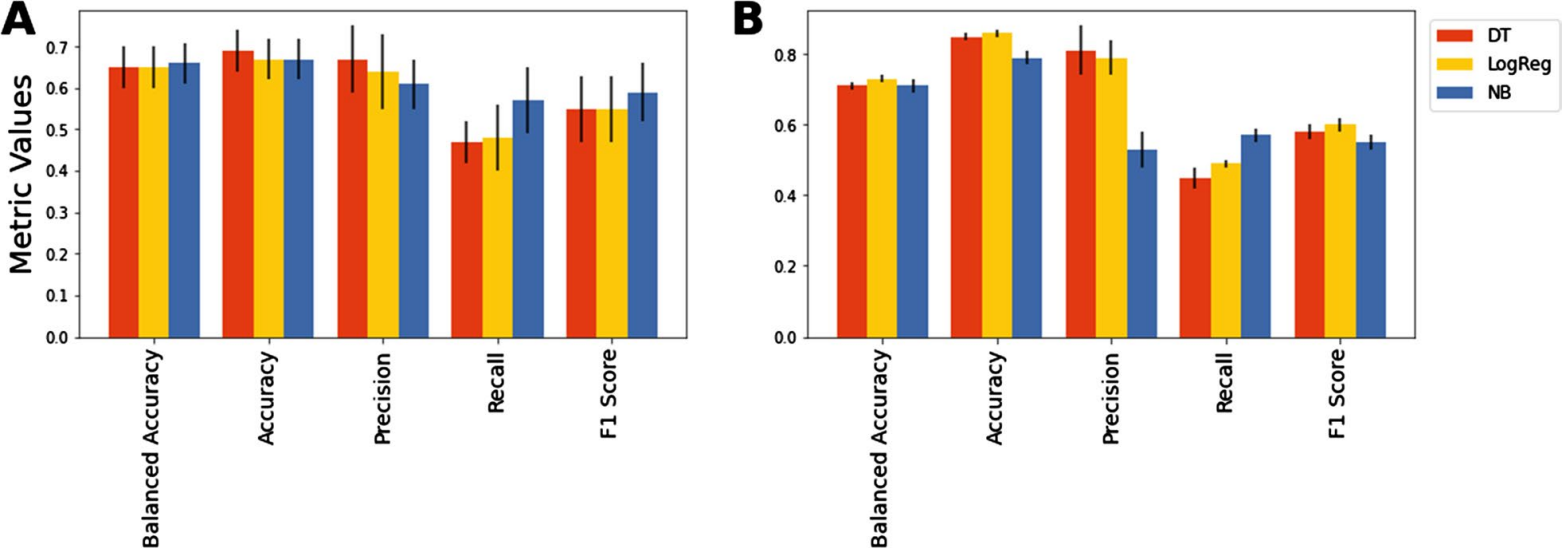
Effect of the choice of base learners on performance of the KBC algorithm. Performance of KBC was tested using decision tree, logistic regression, and Naïve Bayes as the base learners, with data from HIV-1 clade C that describe variability patterns in the high-mannose patch (**A) **and the CD4-binding site (**B**). Error bars indicate standard deviations

The results of the above tests are shown in Fig. 7. For the high-mannose patch (Fig. 7A), decision tree yielded modestly higher point estimates for accuracy and precision, whereas Naïve Bayes showed modestly better recall and F1 score. However, these differences were not statistically significant (see error bars in Fig. 7). Therefore, for this dataset, the choice of base learner did not impact the performance of the KBC algorithm. For the CD4-binding site (Fig. 7B), decision tree and logistic regression performed equally well as the base learners, and were both better than Naïve Bayes in accuracy and precision metrics. Similar to the high-mannose-patch data, the recall was better for Naïve Bayes; however, this improvement was not sufficient to counterbalance the considerably lower precision, resulting in an F1 score for Naïve Bayes that was modestly smaller than that of decision tree and logistic regression.

In summary, KBC is a general algorithm that can apply a wide range of base learners. As shown in Fig. 7, the choice of base learner may affect the performance of the KBC algorithm. These effects are likely specific to each application. In this study, we utilized decision tree as the base learner due to its speed and performance, which was at least as good as other options.

## Discussion

Many viruses, including HIV-1, exhibit high error rates during their replication [61, 62]. New variants of their proteins are continuously generated in the host. The ability to create diversity allows viruses to rapidly adapt to selective pressures, including antiviral therapeutics. The first step in the emergence of resistance is the appearance of sequence variability at a position of the viral protein targeted by the therapeutic. Variability patterns across the Env protein seem random and are thus considered unpredictable. In this study, we examined whether positions that exhibit sequence variability are spatially clustered on the three-dimensional structure of the HIV-1 Env protein. Specifically, we tested whether the absence or presence of sequence variability at any position of Env in a patient can be predicted by variability at adjacent positions on the protein. To address this question, we developed a new dynamic ensemble selection algorithm.

The KBC algorithm defines the neighborhood of a new data point using the KNN algorithm. Specifically, for each position of interest, KBC defines the neighborhood by identifying observations (patient samples) that have a similar feature vector (i.e. a similar variability profile of the 10 adjacent positions). The k-best classifier(s) within that neighborhood are then selected based on a weighted scoring procedure. By comparing each classifier’s score with a minimum acceptance threshold, we obtain the set of best classifiers to predict the class label for each new instance. The dynamism, along with the specific design, resulted in a flexible approach that is not constrained to select a constant number of learners every time that it predicts the class label of a new observation. Based on the performance of the learners, only those classifiers surpassing an explicit expectation are chosen, resulting in an improvement in the overall performance. The novelty of this algorithm is in the dynamic classifier selection mechanism, in which we designed a weighting procedure to evaluate each classifier’s performance within a neighborhood of an instance and decide if the classifier is good enough to classify the observation. This approach is based on bootstrap resampling, which creates out-of-bag samples that can be used along with the resampled data in the classifiers’ evaluation process.

We applied the algorithms to predict the level of variability at individual positions of Env based on variability at adjacent positions on the molecule. Results were compared with a variety of state-of-art methods, such as the Adaboost, Naïve Bayes, logistic regression, linear and quadratic discriminant analysis methods, and SVM. Overall, the KBC algorithm predicted the absence or presence of variability better than the above machine learning tools. Importantly, KBC showed considerable improvement in predicting variability at multi-position features. We tested two Env domains targeted by therapeutics; the CD4-binding site and the high-mannose patch (composed of 23 and 10 positions, respectively). Both domains constitute targets for multiple HIV-1 therapeutics [34, 35, 49–53]. These epitopes were analyzed using sequence data from patients infected by HIV-1 clades B and C, which were tested separately. For both domains and in both clades, the absence or presence of variability was predicted better using KBC than other algorithms. Interestingly, performance varied with the domain of Env tested. Only modest enhancement of performance by the KBC method was observed for the high-mannose patch, whereas dramatic enhancement was observed for the CD4-binding site, with improvement in all critical classification metrics. These results are encouraging since therapeutics do not recognize single positions but rather multi-position footprints on the protein; a change at any position can reduce the binding of the agents and increase clinical resistance [56, 57, 63]. The ability to predict the variability in a given domain based on the adjacent sites suggests that if these associations are stable over time, they may provide insight into future changes that can occur based on the current patterns of variability in the patient. Such knowledge can be applied to personalize therapeutics based on the likelihood for resistance mutations to appear in each patient. Notably, for small datasets (e.g. analysis of single Env positions), KBC exhibited high point estimates but also high standard deviations. By contrast, using larger datasets (e.g. multi-position targets), KBC exhibited both higher estimates and also smaller standard deviations compared to other algorithms. This finding suggested that KBC is more suitable for large datasets.

We observed that despite using homogenous and simple learners, KBC competes well with even sophisticated algorithms such as SVM, Adaboost, and discriminant analysis techniques. We also evaluated the effects of using logistic regression and Naïve Bayes as the base learners. Our results suggested that the choice of base learner may impact the overall performance; however, the effects are likely specific for each problem. We selected to focus our studies on decision tree as the base learner because of its relative speed and its performance, which was at least as high as that of the other options. Nevertheless, we note that by using more advanced methods as the base learner and by increasing diversity using a pool of different methods, KBC may exhibit even higher performance, which can be explored in future studies.

Our study is subjected to a few limitations which suggest future research directions. First, in the current design, the entire training dataset is scanned for each new instance to find the neighbors using KNN. This may lead to computational intractability for very large datasets. Innovative methods for defining the neighborhood can be applied to improve efficiency, such as clustering algorithms that group similar instances [26]. In this manner, the most similar cluster to the new data point can be identified, and performance of the classifiers is evaluated within that isolated “neighborhood”. Second, for small datasets, KBC often shows higher classification metrics than other methods but also higher standard deviations. We anticipate that the use of more sophisticated base learners will reduce this variance. Nevertheless, it should be noted that the use of new learners will likely require an additional optimization phase to balance the running time of the algorithm with classification performance.

## Conclusions

To better understand the patterns of amino acid variability across the Env protein in HIV-1-infected patients, we developed a new classification algorithm based on dynamic ensemble selection. This algorithm, designated k-best classifiers (KBC), accurately predicts the absence or presence of variability at Env positions and at multi-position epitopes based on the variability at adjacent positions on the three-dimensional structure of the protein. The primary advantage of KBC is that it does not use the same set of classifiers for the entire problem space; instead, it identifies the subset of base learners that are capable of better predicting class labels for the new observations based on their neighborhood. This flexibility helps to avoid the loss of helpful learners and to limit the retention of weak learners that occurs when a fixed number of classifiers is used. We applied KBC to individual positions as well as multi-position epitopes that are commonly targeted by antibodies elicited by HIV-1 infection. KBC showed superior performance in predicting variability patterns at these sites relative to the base learner and a large panel of classification techniques we tested. Higher point estimations and lower standard deviations of the estimates were observed. This study supports the notion that positions with sequence variability in each patient are spatially clustered on the three-dimensional structure of the Env protein. This knowledge and the algorithm developed here can be applied to refine models aimed at predicting future changes in viral proteins within the host as the basis for personalizing antiviral therapeutics.

## Data Availability

All datasets used in this study can be downloaded from: https://github.com/haimlab/KBC-Manuscript-Data, including: (i) Matrices that describe the distances (in Å) between the closest atoms of any two Env residues on the cryo-EM structures of clade B Env JRFL (PDB ID 5FUU) or clade C Env 426c (PDB ID 6MZJ). (ii) Amino acid variability at all 856 Env positions for 191 clade B patients and 109 clade C patients.

## Abbreviations

ADA: Adaboost
DES: Dynamic ensemble selection
DT: Decision tree
Env: Envelope glycoprotein
HIV-1: Human immunodeficiency virus type 1
KBC: K best classifiers
KNN: K-nearest neighbors
LDA: Linear discriminant analysis
LogReg: Logistic regression
NB: Naïve Bayes
OOB: Out-of-bag sample
QDA: Quadratic discriminant analysis
RF: Random forest
SVM: Support vector machine
